# Effects of Acute Caffeine Ingestion on Repeated Sprint Ability: A Systematic Review and Meta-Analysis

**DOI:** 10.3390/nu17213475

**Published:** 2025-11-05

**Authors:** Yunteng Wang, Wantang Su, Shiyan Zhang, Li Zhao, Yuanyuan Lv, Boya Gu, Laikang Yu

**Affiliations:** 1 Beijing Key Laboratory of Sports Performance and Skill Assessment, Beijing Sport University, Beijing 100084, China; 2025110097@bsu.edu.cn (Y.W.); zhaolispring@126.com (L.Z.); sunflowerlyy@bsu.edu.cn (Y.L.); 2 Department of Exercise Physiology, Beijing Sport University, Beijing 100084, China; wantang1999@126.com (W.S.); 17634977464@163.com (S.Z.); 3 China Institute of Sport and Health Science, Beijing Sport University, Beijing 100084, China; 4 Beijing Anti-Doping Laboratory, Beijing Sport University, Beijing 100084, China; 5 Department of Strength and Conditioning Assessment and Monitoring, Beijing Sport University, Beijing 100084, China

**Keywords:** caffeine, repeated sprint ability, ergogenic aid, high-intensity exercise

## Abstract

**Background/Objectives**: Caffeine is widely recognized as an ergogenic aid, yet evidence regarding its acute effects on repeated sprint ability (RSA) remains inconsistent. This systematic review and meta-analysis aimed to evaluate the effects of acute caffeine ingestion on RSA across different populations, exercise modalities, and dosage levels. **Methods**: A comprehensive literature search was conducted in the PubMed, EBSCO, Cochrane Library, Web of science, and Scopus databases. Data were pooled using the weighted mean difference (WMD) with 95% confidence interval (CI). **Results**: Thirteen studies met the inclusion criteria. Acute caffeine ingestion significantly enhanced RSA peak power output (PPO) compared with placebo (WMD, 5.28; 95% CI, 2.49 to 8.07; *p* = 0.0002). Subgroup analyses revealed significant improvements in both males (WMD, 13.11; 95% CI, 5.63 to 20.59; *p* = 0.0006) and females (WMD, 4.03; 95% CI, 1.10 to 6.97; *p* = 0.007). A caffeine dose of ≥6 mg/kg body weight (BW) produced greater ergogenic benefits (WMD, 6.67; 95% CI, 3.32 to 10.02; *p* < 0.0001) than lower doses (WMD, 2.16; 95% CI, −2.87 to 7.19; *p* = 0.40). Moreover, a more pronounced enhancement was observed in cycling-based RSA (WMD, 8.77; 95% CI, 1.98 to 15.56; *p* = 0.01) compared with running-based protocols (WMD, 4.56; 95% CI, 1.58 to 7.55; *p* = 0.003). **Conclusions**: Acute caffeine ingestion significantly enhances RSA, particularly at doses ≥6 mg/kg BW. This effect is consistent across both male and female participants, with no statistically significant sex difference observed in the pooled analysis. These findings reinforce caffeine’s role as an effective ergogenic aid for optimizing high-intensity intermittent performance, with the strongest benefits evident in cycling exercise.

## 1. Introduction

Caffeine, the primary bioactive compound found in widely consumed beverages such as coffee, tea, and cocoa, acts as a central nervous system stimulant and adenosine receptor antagonist [1]. It is rapidly absorbed and exerts diverse physiological effects, including reducing fatigue, enhancing lipolysis, and increasing alertness [2,3,4]. Although generally considered safe at typical consumption levels, excessive intake may cause adverse effects such as anxiety, insomnia, and gastrointestinal discomfort [5,6,7]. Owing to its pronounced physiological actions, caffeine has long been a subject of extensive research in the context of performance enhancement and remains one of the most widely used legal psychoactive substances worldwide.

In the field of sports science, caffeine is recognized as a potent ergogenic aid capable of augmenting athletic performance through multiple mechanisms. Early studies demonstrated that caffeine enhances aerobic endurance primarily by promoting fat oxidation and conserving muscle glycogen stores [8,9]. Subsequent research extended these observations, indicating that caffeine may also improve anaerobic endurance and high-intensity exercise performance through multiple mechanisms, including reduced pain perception, enhanced muscle contractility, and improved neural drive [10,11]. Notably, caffeine was only removed from the World Anti-Doping Agency’s list of prohibited substances in 2004, thereby legitimizing its use as an ergogenic aid among elite athletes. Since then, caffeine-containing energy drinks have emerged as a popular vehicle for delivering this ergogenic aid. These beverages typically combine substantial amounts of caffeine with additional ingredients such as carbohydrates, B vitamins, and taurine, thereby providing an auxiliary energy source during exercise [12,13]. However, the primary ergogenic benefits are largely attributed to their high caffeine content, with nearly half of professional athletes reportedly using caffeine-based supplements to enhance performance [14]. According to the International Society of Sports Nutrition, caffeine consumption approximately 60 min prior to exercise can effectively improve various aspects of athletic performance, including endurance capacity, high-intensity effort, and muscular strength [15].

Repeated sprint ability (RSA) is a performance parameter that depends primarily on the adenosine triphosphate-creatine phosphate (ATP-CP) energy system and plays a crucial role in high-intensity intermittent sports such as basketball, soccer, and rugby [16,17,18,19]. However, the ergogenic potential of acute caffeine ingestion for improving RSA remains controversial. Several studies have reported that acute caffeine supplementation enhances RSA by increasing both peak power output (PPO) and mean power output (MPO) [20,21]. In contrast, other studies have failed to detect such benefits. For instance, Bernardo et al. [22] observed no significant differences in PPO or MPO between caffeine and placebo conditions in male participants. Similarly, Houda et al. [23] reported that an acute caffeine dose of 3 mg/kg body weight (BW) did not significantly influence RSA in young female athletes, whereas higher doses of 6 or 9 mg/kg BW improved performance by reducing both the fastest and mean sprint time, suggesting a potential dose–response effect. Conversely, another research demonstrated that even a 3 mg/kg BW caffeine dose was sufficient to enhance both PPO and MPO during repeated sprints [24]. Collectively, these inconsistent findings indicate that the ergogenic effects of caffeine on RSA remain equivocal and may be influenced by interindividual differences in caffeine sensitivity [25].

Existing meta-analyses examining the effects of acute caffeine ingestion on RSA have also produced divergent conclusions. For instance, Gomez-Bruton et al. [26] reported no significant effect of caffeine on RSA in females athletes, whereas López-Torres et al. [27] demonstrated a positive ergogenic effect. Similarly, Lopes-Silva et al. [28] found that acute caffeine ingestion did not significantly improve RSA, while another meta-analysis revealed that caffeine markedly enhanced high-intensity performance in elite athletes during both real or simulated competitions [29]. These discrepancies among meta-analytic findings may stem from methodological limitations, such as the inclusion of only female participants, the absence of dosage stratification, and inadequate control for habitual caffeine consumption.

Therefore, to address these inconsistencies and methodological gaps, the present systematic review and meta-analysis aimed to comprehensively evaluate the effects of acute caffeine ingestion on RSA.

## 2. Materials and Methods

### 2.1. Design

This systematic review and meta-analysis was conducted in accordance with the Preferred Reporting Items for Systematic Evaluation and Meta-Analysis (PRISMA) guidelines [30]. The study protocol was prospectively registered in the International Prospective Register of Systematic Reviews (PROSPERO; registration number CRD420251150247).

### 2.2. Search Strategy

A comprehensive literature search was performed in PubMed, EBSCO, Cochrane Library, Web of science, and Scopus databases from their inception to 6 September 2025. The search strategy employed the keywords and MESH terms “caffeine” and “repeated sprint ability” (Table S1). Additionally, reference lists of the identified systematic reviews and meta-analyses were manually searched to identify potential eligible studies. Article screening and selection were independently conducted by two authors (Y.W. and W.S.).

### 2.3. Eligibility Criteria

The inclusion criteria were established based on the Population, Intervention, Comparison, and Outcome (PICO) principle: (1) Population: human participants; (2) Intervention: administration of acute, rather than long-term, caffeine interventions; (3) Comparison: evaluation of acute effects between caffeine ingestion and placebo control groups; and (4) Outcome: the primary endpoint was PPO.

PPO was assessed using cycle ergometer, while fastest sprint time was measured during running-based tests. Where necessary, fastest sprint time data were converted to PPO using the following equation [31]:

Power output = (body mass (kg) × distance (m)^2^)/time (s)^3^ (1)

Exclusion criteria were as follows: (1) non-English publications; (2) conference papers; (3) review articles; and (4) animal or in vitro studies.

### 2.4. Data Extraction

Data extraction was independently conducted by two authors (Y.W. and W.S.) using a predefined template. The extracted information included: (1) study characteristics (first author, publication year, country); (2) subject characteristics (age, sex, sample size); (3) intervention characteristics (caffeine dosage and timing); and (4) outcome characteristics (exercise modality, PPO, fastest sprint time).

### 2.5. Methodological Quality Assessment

The methodological quality of the included studies was evaluated using the Cochrane risk of bias tool [1,32] and further evaluated with the Physiotherapy Evidence Database (PEDro) scale. The Cochrane risk of bias tool evaluates five domains of potential bias, including selection bias, performance bias, detection bias, attrition bias, and reporting bias. Each domain rated as having a low, unclear, or high risk of bias. The PEDro scale comprises 11 items, and studies scoring <4 points, 4–5 points, 6–8 points, and ≥9 points were categorized as poor, average, good, and excellent quality, respectively [33].

### 2.6. Certainty Assessment

The Grading of Recommendations Assessment, Development and Evaluation (GRADE) approach was used to assess the certainty of evidence across outcomes, with ratings categorized as high, moderate, low, or very low. The GRADE assessment was conducted two independent authors (Y.W. and W.S.).

### 2.7. Statistical Analysis

From each included study, the mean and standard deviation (SD) of RSA-related variables were extracted for both caffeine and placebo conditions. Data were pooled using fixed-effects models to obtain the weighted mean difference (WMD) and 95% confidence intervals (CIs). When studies reported standard errors (SEs) and 95% CIs instead of SDs, the corresponding SD was estimated [34,35].

Heterogeneity was assessed using the *I*^2^ statistic, in cases of substantial heterogeneity, subgroup analysis and sensitivity analysis were conducted to explore potential sources of variation [36,37]. Subgroup analyses examined the influence of (1) caffeine dosage (<6 vs. ≥6 mg/kg BW), (2) exercise modality (cycling vs. running), and (3) participant sex (male vs. female).

Forest plots were generated using RevMan software (Version 5.4), while sensitivity analysis, funnel plot, and meta-regression were performed using Stata software (Version 15.0). Statistical significance was set at *p* < 0.05.

## 3. Results

### 3.1. Studies Selection

**Figure 1:**
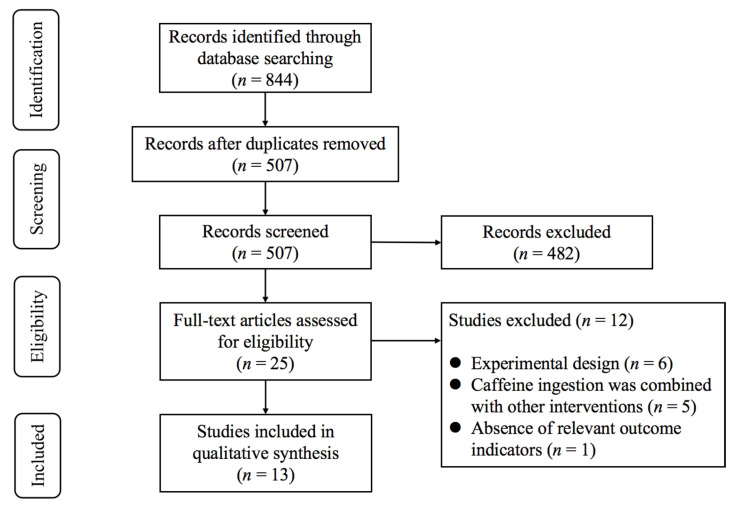
PRISMA flowchart of study selection.

As illustrated in Figure 1, the initial database search identified 844 records. After removing duplicates, 507 studies remained for title and abstract screening. Following this process, 25 potentially eligible studies were retained for full-text evaluation. Upon detailed review, 12 studies were excluded for the following reasons: (1) experimental design (*n* = 6); (2) caffeine ingestion was combined with other interventions (*n* = 5); (3) absence of relevant outcome indicators (*n* = 1). Finally, 13 studies [10,11,12,13,14,29,30,31,32,33,34,35,36] met the inclusion criteria.

### 3.2. Characteristics of the Included Studies

The characteristics of the included studies and participants are summarized in Table 1. Among the 13 studies, 2 studies [38,39] employed a low caffeine dosage (<6 mg/kg BW), 7 studies [21,22,40,41,42,43,44] used a high caffeine dosage (≥6 mg/kg BW), and 4 studies [20,23,24,45] examined both low and high dosages. Regarding participant characteristics, six studies [21,22,24,38,42,44] involved only males, 6 studies [20,23,40,41,43,45] included only females, and 1 study [39] involved participants of both sexes. Eight studies [21,22,24,38,39,42,43,45] reported PPO data assessed via cycling tests, whereas 5 studies [20,23,40,41,44] provided data for fastest sprint time measured using running tests. Caffeine dosage ranged from 3 to 9 mg/kg BW, with ingestion typically occurring 60 min prior to testing.

### 3.3. Risk of Bias

The risk of bias assessment using the Cochrane risk of bias tool identified two studies as having a high risk of reporting bias, while the remaining studies were generally classified as low risk across the domains of selection, performance, detection, attrition, and reporting biases (Figure S1). Based on the PEDro scale, 13 studies [20,21,22,23,24,38,39,40,41,42,43,44,45] were rated as excellent quality (Table S2).

### 3.4. Meta-Analysis

**Figure 2:**
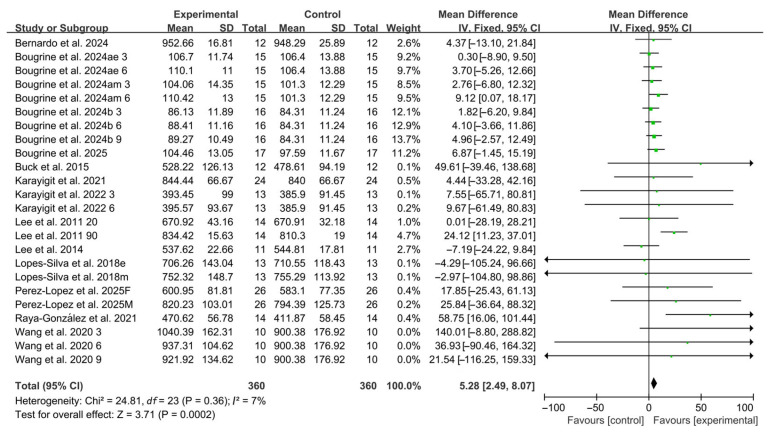
Meta-analysis results of the effects of acute caffeine ingestion on RSA [20,21,22,23,24,38,39,40,41,42,43,44,45]. Articles by the same author are distinguished by the suffixes “a” and “b”. Morning and evening sessions are denoted by “m” and “e”, respectively; participant sex by “M” (male) and “F” (female); caffeine dose by “3”, “6”, or “9” (mg/kg body weight); and inter-interval rest duration by “20” or “90” (min).

Compared with placebo, acute caffeine ingestion significantly enhanced RSA (WMD, 5.28, 95% CI, 2.49 to 8.07, *p* = 0.0002, *I*^2^ = 7%, Figure 2).

### 3.5. Subgroup Analysis

#### 3.5.1. Caffeine Dosage

**Figure 3:**
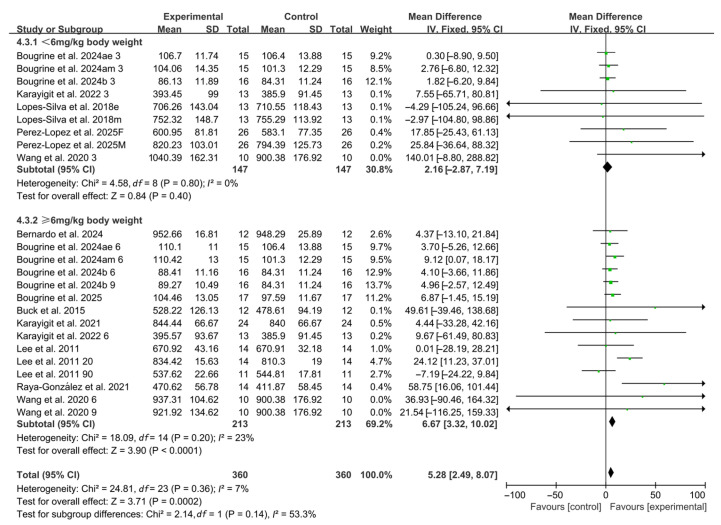
Meta-analysis results of the effects of caffeine dosage on RSA [20,21,22,23,24,38,39,40,41,42,43,44,45]. Articles by the same author are distinguished by the suffixes “a” and “b”. Morning and evening sessions are denoted by “m” and “e”, respectively; participant sex by “M” (male) and “F” (female); caffeine dose by “3”, “6”, or “9” (mg/kg body weight); and inter-interval rest duration by “20” or “90” (min).

When stratified by caffeine dosage, 9 studies investigated low doses (<6 mg/kg BW), including 7 studies using 3 mg/kg BW and 2 studies using 5 mg/kg BW. No significant improvements in RSA were observed within this subgroup (WMD, 2.16, 95% CI, −2.87 to 7.19, *p* = 0.40, *I*^2^ = 0%, Figure 3). In contrast, 15 studies assessed high doses, with 13 studies using 6 mg/kg BW and 2 studies using 9 mg/kg BW. In this subgroup, caffeine ingestion produced a significant improvement in RSA (WMD, 6.67, 95% CI, 3.32 to 10.02, *p* < 0.0001, *I*^2^ = 23%, Figure 3).

#### 3.5.2. Exercise Modality

**Figure 4:**
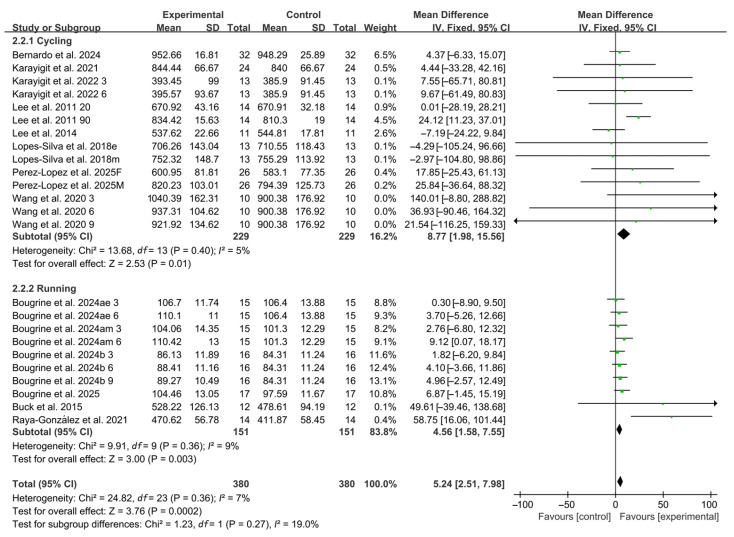
Meta-analysis results of the effects of exercise modality on RSA [20,21,22,23,24,38,39,40,41,42,43,44,45]. Articles by the same author are distinguished by the suffixes “a” and “b”. Morning and evening sessions are denoted by “m” and “e”, respectively; participant sex by “M” (male) and “F” (female); caffeine dose by “3”, “6”, or “9” (mg/kg body weight); and inter-interval rest duration by “20” or “90” (min).

When stratified by exercise modality, 14 studies employed cycling protocols and 10 studies used running-based tests. Significant improvements in RSA were observed for both modalities (cycling: WMD, 8.77, 95% CI, 1.98 to 15.56, *p* = 0.01, *I*^2^ = 5%; running: WMD, 4.56, 95% CI, 1.58 to 7.55, *p* = 0.003, *I*^2^ = 9%, Figure 4), with running-based RSA exhibiting a more pronounced effect.

#### 3.5.3. Participant Sex

**Figure 5:**
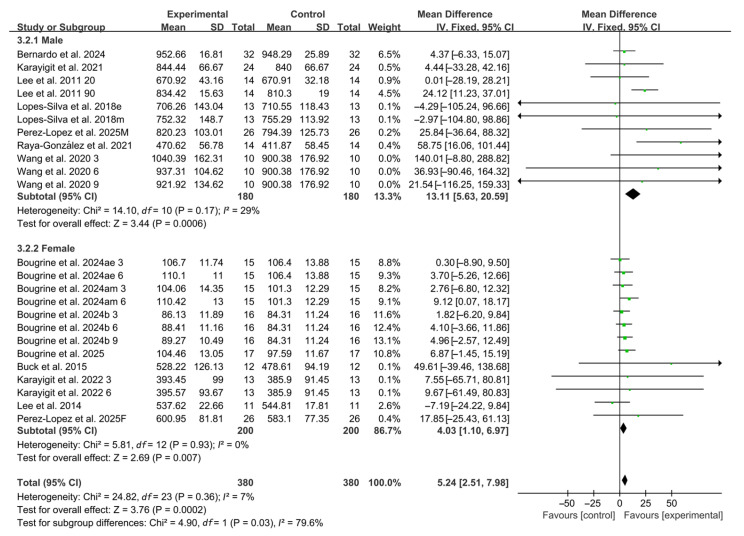
Meta-analysis results of the effects of participant sex on RSA [20,21,22,23,24,38,39,40,41,42,43,44,45]. Articles by the same author are distinguished by the suffixes “a” and “b”. Morning and evening sessions are denoted by “m” and “e”, respectively; participant sex by “M” (male) and “F” (female); caffeine dose by “3”, “6”, or “9” (mg/kg body weight); and inter-interval rest duration by “20” or “90” (min).

Subgroup analyses by participant sex revealed that caffeine ingestion significantly enhanced RSA in both male and female (male: WMD, 13.11, 95% CI, 5.63 to 20.59, *p* = 0.0006, *I*^2^ = 29%; female: WMD, 4.03, 95% CI, 1.10 to 6.97, *p* = 0.007, *I*^2^ = 0%, Figure 5), with male participants exhibiting a more pronounced effect.

### 3.6. Publication Bias

Possible publication bias was assessed using funnel plot analysis (Figure S2). Visual inspection indicated a substantial asymmetry, suggesting a likelihood of publication bias. However, application of the Duval and Tweedie trim-and-fill method did not alter the overall effect size, confirming the robustness of the pooled estimates. This finding was supported by Egger’s test (*p* = 0.328, Table S3).

### 3.7. Sensitivity Analysis

Sensitivity analysis demonstrated that the overall effect of acute caffeine ingestion on RSA remained stable in both magnitude and direction after sequential exclusion of individual studies (Figure S3). These results confirm the robustness and reliability of the meta-analytic findings.

### 3.8. GRADE Summary

The certainty of the evidence underpinning our findings was systematically assessed using the GRADE framework. Following this rigorous appraisal, the overall certainty of the evidence was rated as high, indicating strong confidence in the reliability and generalizability of our conclusions (Table S4).

## 4. Discussion

### 4.1. Main Findings

The primary objective of this study was to systematically synthesize and quantify the effects of acute caffeine ingestion on RSA across diverse study populations, exercise modalities, and caffeine dosages. A total of 13 studies were included in the final analysis, and the pooled results revealed that acute caffeine ingestion exerted a statistically significant ergogenic effect on RSA in both male and female participants. Subgroup analyses further indicated that this performance-enhancing effect was consistent across two common exercise modalities—cycling and running. Notably, a dosage-dependent response was observed, with caffeine ingestion at a dose of ≥6 mg/kg BW yielding a more pronounced ergogenic impact on RSA compared to lower doses.

### 4.2. Effects of Acute Caffeine Ingestion on Repeated Sprint Ability

The findings of the present study demonstrated that acute caffeine ingestion significantly improved RSA, which is congruent with the results of prior studies [27,29]. Beyond oral ingestion, alternative routes of caffeine administration have also been explored for their effects on anaerobic performance. For instance, caffeine mouth rinse (without swallowing) has been shown to enhance maximal power production during short-duration, high-intensity exercise. This effect is thought to be mediated by caffeine’s activation of oral cavity adenosine receptors, which transmit sensory signals to the central nervous system to augment motor unit recruitment, a mechanism that may also contribute to improvements in RSA, given the sport-specific demand for repeated maximal power outputs [46].

Previous research has posited that ergogenic efficacy of caffeine may be attenuated under conditions of cumulative fatigue, a physiological challenge inherently associated with RSA protocols [38]. Mechanistically, caffeine exerts its central and peripheral effects primarily as a non-selective adenosine receptor antagonist, specifically targeting A1 and A2A receptors [1]. By competitively binding to these receptors, caffeine inhibits the action of adenosine, a key neuromodulator of fatigue, sleep, and arousal, thereby delaying the onset of perceived fatigue and enhancing central drive [47]. It is important to distinguish between caffeine’s effects on alertness and its capacity to reverse physiological fatigue: while caffeine has been shown to transiently improve subjective ratings of alertness in sleep-deprived individuals, it cannot mitigate the underlying physiological deficits (e.g., reduced glycogen stores, impaired neuromuscular function) induced by prolonged sleep deprivation [48]. Extending this to exercise contexts, caffeine ingestion has been shown to reduce time to exhaustion during endurance exercise; however, this effect was attributed to improvements in mood and perceived exertion rather than a direct attenuation of neuromuscular fatigue [49]. Consistent with these findings, further research has indicated that the efficacy of caffeine in enhance RSA diminishes as exercise-induced fatigue accumulates over successive sprints [50]. To address this potential attenuation and ensure sensitivity to caffeine’s true ergogenic potential, the present study focused on two key RSA outcomes: PPO (a measure of maximal anaerobic capacity) and fastest sprint time (a marker of optimal sprint performance) rather than MPO and mean sprint time, which are more susceptible to the confounding effects of cumulative fatigue.

A critical methodological consideration in caffeine research is the timing of RSA assessment relative to ingestion, as plasma caffeine concentration reaches its peak approximately 60 min post-ingestion in healthy adults [51]. To minimize variability in caffeine bioavailability across studies, all 13 included investigations consistently administered caffeine 60 min prior to RSA testing, a methodological consistency that strengthens the internal validity of the present meta-analysis.

### 4.3. Effects of Caffeine Dosage on Repeated Sprint Ability

Caffeine dosage is a critical moderator of its ergogenic effect on RSA, and the present subgroup analysis confirmed a dose–response relationship. Specifically, acute caffeine ingestion at a dose of ≥6 mg/kg BW (high-dose) significantly enhanced RSA, while acute caffeine ingestion of <6 mg/kg BW (low-dose) did not elicit a statistically significant effect. This observation aligns with previous studies that high-dose caffeine confers a robust ergogenic benefit for RSA [23,52]. Interestingly, existing literature suggests that doses exceeding 6 mg/kg BW (e.g., 9 mg/kg BW) do not further amplify RSA improvements; studies comparing 6 mg/kg BW and 9 mg/kg BW have found no significant differences in RSA outcomes, indicating a potential ceiling effect for caffeine’s ergogenic action on repeated sprints [23,52].

A notable discrepancy emerged between the present findings and a study by Wang et al. [24], which reported that low-dose (rather than high-dose) caffeine improved RSA. This inconsistency may be attributed to the unique characteristics of the participant sample in Wang et al.’s study: the authors recruited individuals with low habitual caffeine consumption (defined as <60 mg/day). This may Population-based research has established that habitual caffeine intake modulates sensitivity to acute caffeine ingestion—low consumers typically exhibit greater physiological and performance responses to lower doses, whereas high consumers may develop tolerance, requiring higher doses to achieve similar effects [53]. Thus, the low habitual intake of Wang et al.’s participants likely enhanced their responsiveness to low-dose caffeine, accounting for the divergent results.

### 4.4. Effects of Exercise Modality on Repeated Sprint Ability

Cycling and running are the two most prevalent exercise modalities used to assess RSA in experimental research, as they allow for precise measurement of power output (cycling) and sprint time (running) while controlling for external variables (e.g., terrain, technique). The present subgroup analysis confirmed that acute caffeine ingestion improved RSA across both modalities, with running-based RSA exhibiting a more pronounced effect, a finding that is supported by multiple previous studies [46,54,55]. In cycling-based RSA protocols, Schneiker et al. [54] reported that participants who ingested caffeine (6 mg/kg BW) exhibited significantly higher PPO across repeated sprints compared to those in the placebo group, a result that was later replicated by Beaven et al. [46], who observed a significant increase in PPO following caffeine ingestion in trained males. In running-based RSA tests, Glaister et al. [55] demonstrated that acute caffeine ingestion (5 mg/kg BW) significantly reduced fastest sprint time in physically active males.

An important practical consideration is the ecological validity of exercise modalities for specific sports. Running-based RSA tests entail greater total energy expenditure and neuromuscular demand (e.g., activation of lower-body muscles, balance control) compared to cycling tests, which are constrained by fixed pedaling mechanics [56]. This difference suggests that cycling may not be the optimal modality for assessing RSA in running-based sports (e.g., basketball, volleyball, soccer), as it fails to replicate the movement patterns and physiological stressors inherent to these sports. This potential lack of ecological validity may explain why some studies utilizing cycling tests to assess RSA in running-based sports athletes found no significant effect of acute caffeine ingestion on PPO [57,58]. These findings highlight the importance of selecting exercise modalities that align with the sport-specific demands of the participant population when investigating caffeine’s effects on RSA.

### 4.5. Effects of Participant Sex on Repeated Sprint Ability

While the present meta-analysis confirmed that acute caffeine ingestion significantly improved RSA in both males and females, subgroup analysis and a synthesis of existing literature revealed substantial heterogeneous in the magnitude and consistency of this effect across sexes.

In the male group, the pooled results indicated a significant ergogenic effect of caffeine on RSA; however, this finding contrasts with a study by Kopec et al. [59], which reported no differences in RSA between caffeine (6 mg/kg BW) and placebo groups in male team-sport athletes. The discrepancy may be attributed to the low sample size (*n* = 11) and moderate training status of participants in Kopec et al.’s study, which may have reduced statistical power to detect small-to-moderate effect sizes. In the female subgroup, our findings align with previous studies [23,60]. Bougrine et al. [23] demonstrated that acute caffeine ingestion at 6 mg/kg BW significantly improved RSA in female team-sport athletes, while another study by Lara et al. [60] reported that even a low dose of caffeine (3 mg/kg BW) enhanced RSA in female soccer players. Conversely, a previous meta-analysis by Gomez-Bruton et al. [26] found no significant effect of acute caffeine ingestion on RSA in female team-sport athletes. This null finding may be attributable to methodological heterogeneity, particularly the restriction of their analytical sample to participants aged ≥ 18 years, thereby limiting direct comparability with investigations incorporating younger cohorts.

A key physiological mechanism underlying potential sex differences in caffeine responsiveness is the rate of caffeine metabolism. Females have been shown to metabolize caffeine more slowly than males, primarily due to the inhibitory effect of estrogen on CYP1A2, the primary hepatic enzyme responsible for caffeine degradation [61]. This slower metabolism results in prolonged plasma caffeine concentrations in females, which may alter the timing and magnitude of caffeine’s ergogenic effect on RSA. Pérez-López et al. [39] provided empirical support for this mechanism, reporting that following acute caffeine ingestion, males exhibited higher PPO in the first two sprints of an RSA protocol, whereas females demonstrated superior PPO in the final two sprints. This temporal difference likely reflects the prolonged caffeine availability in females, which mitigates fatigue during later sprints.

Given the equivocal nature of existing evidence, with some studies reporting sex-specific effects and others finding no differences, further research is warranted to explicitly investigate sex as a moderator of caffeine’s effects on RSA. Future studies should control for variables such as menstrual cycle phase (in females), habitual caffeine intake, and training status to reduce heterogeneity and clarify the role of sex.

### 4.6. Practical Implications

These findings have practical implications for athletes and coaches seeking to optimize RSA performance: acute caffeine ingestion at 6 mg/kg BW, administered 60 min pre-exercise, represents an evidence-based strategy to enhance repeated sprint outcomes. Future research should address the limitations of the present study by explicitly investigating the moderating effects of training status, habitual caffeine intake, and participant age on caffeine’s ergogenic effect on RSA. Additionally, studies utilizing sport-specific RSA protocols (e.g., soccer-specific running drills, basketball agility sprints) are needed to further enhance the ecological validity and translational utility of caffeine research in applied sports settings.

### 4.7. Limitations

This study has several limitations. First, previous studies have employed diverse forms of caffeine administration (e.g., powder, capsules, mouth rinse, caffeinated beverages) [39,41,62,63]. Due to the limited number of eligible studies, the present analysis included only powder and capsule forms without distinguishing their effects separately. Second, substantial variability in the athletic capabilities of participants may have influenced RSA outcomes following acute caffeine ingestion. Differences in training status, habitual caffeine use, and sport-specific demands can substantially modulate the ergogenic response to caffeine [64,65]. As previously noted, cycling may not represent the most appropriate testing modality for RSA in running-based sports [56]. Therefore, future research should further delineate the effects of acute caffeine ingestion on RSA across various sport types and performance contexts.

Finally, although several studies have reported adverse effects associated with acute caffeine ingestion (e.g., such as ventricular fibrillation, irritability, and nervousness) [23,66], these outcomes were not analyzed in the current study. For example, Bougrine et al. [23] observed a higher incidence of side effects following caffeine ingestion at a dose of 9 mg/kg BW. Consequently, future investigations should consider not only the ergogenic potential but also the safety profile of different caffeine dosages to optimize performance benefits while minimizing potential risks. In line with recommendations from the International Society of Sports Nutrition (ISSN), caffeine doses of 3–6 mg/kg BW can enhance exercise performance, whereas doses of 9 mg/kg are associated with a higher incidence of adverse effects [15].

## 5. Conclusions

The present systematic review and meta-analysis provide robust evidence that acute caffeine ingestion (administered 60 min prior to exercise) exerts a significant ergogenic effect on RSA in healthy adults. This effect is consistent across both male and female participants, with no statistically significant sex difference observed in the pooled analysis. Furthermore, acute caffeine ingestion enhances RSA in both cycling and running exercise modalities, though the ecological validity of cycling for running-based sports should be considered when interpreting these results. Moreover, caffeine ingestion at ≥6 mg/kg BW yields a more pronounced ergogenic impact on RSA, whereas doses < 6 mg/kg BW do not elicit significant performance improvements.

## Figures and Tables

**Table 1:** Characteristics of the studies included in this meta-analysis.

Study	Country	Sample Size	Age (y)	Caffeine Dose (mg/kg BW)	Timing of Caffeine Ingestion (min)	Exercise Mode	Sprint Protocol	Main Outcomes
Bernardo et al. 2024 [22]	Brazil	12 M	26 ± 4	6	60	Cycling	12 × 6 s; 60 s rest	PPO →
Bougrine et al. 2024 [20]	Tunisia	15 F	18.3 ± 0.5	3, 6	60	Running	6 × (2 × 12.5 m) shuttle sprints; 20 s rest	Morning: FST ↓; Evening: FST →
Bougrine et al. 2024 [23]	Tunisia	16 F	16.9 ± 0.6	3, 6, 9	60	Running	6 × (2 × 12.5 m) shuttle sprints; 20 s rest	FST ↓ in CAF-6 and -9; FST → in CAF-3
Bougrine et al. 2025 [40]	Tunisia	17 F	16.7 ± 0.4	6	60	Running	6 × (2 × 12.5 m) shuttle sprints; 20 s rest	FST ↓
Buck et al. 2015 [41]	Australia	12 F	25.5 ± 1.9	6	60	Running	6 × 20 m; 25 s rest (only set 1)	FST →
Karayigit et al. 2021 [42]	Turkey	24 M	22 ± 1.5	6	60	Cycling	12 × 4 s; 90 s rest	PPO ↑
Karayigit et al. 2022 [45]	Turkey	13 F	20 ± 1	3, 6	60	Cycling	12 × 4 s; 20 s rest	PPO ↑
Lee et al. 2011 [21]	China	14 M	18.7 ± 0.8	6	60	Cycling	2 sets of 12 × 4 s; 20 s or 90 s rest between sprint, 4 min rest between sets	20 s recovery interval: PPO →; 90 s recovery interval: PPO ↑
Lee et al. 2014 [43]	China	11 F	21.3 ± 1.2	6	60	Cycling	10 sets of 5 × 4 s; 20 s active recovery (60–70 rpm, 50 watts)	PPO →
Lopes-Silva et al. 2018 [38]	Brazil	13 M	26.4 ± 4	5	60	Cycling	10 × 6 s; 30 s rest	PPO →
Perez-Lopez et al. 2025 [39]	Spain	26 M and 26 F	24 ± 4.5	3	60	Cycling	4 × 30 s; 90 s rest	PPO: Both sex: Wt1 & 3 & 4 ↑, Wt2 →. Male: Wt1 & 2 ↑, Wt3 & 4 →. Female: contrary to males.
Raya-González et al. 2021 [44]	Spain	14 M	21 ± 2	6	60	Running	5 × 30 m; 30 s rest	FST ↑
Wang et al. 2020 [24]	China	10 M	20.88 ± 2.72	3, 6, 9	60	Cycling	4 × (15 × 5 s); 55 s rest	PPO ↑ in CAF-3; PPO → in CAF-6 and -9

**Note:** y, year; BW, body weight; rpm, rotation per minute; M, male; F, female; FST, fastest sprint time; PPO, peak power output; →, no significant change; ↑, increase; ↓, decrease.

## Data Availability

All data generated or analyzed during this study are included in the article/Supplementary Materials.

## Supplementary Materials

The following supporting information can be downloaded at: https://www.mdpi.com/article/10.3390/nu17213475/s1, Figure S1: Results of Cochrane risk of bias tool; Figure S2: Funnel plot; Figure S3: Sensitivity analysis results; Table S1: Search strategies; Table S2: Characteristics of the studies included in this meta-analysis; Table S3: Methodological assessment of randomized controlled trials included in the systematic review using the PEDro scale; Table S4: Results of Egger’s test; PRISMA 2020 checklist.

## Abbreviations

The following abbreviations are used in this manuscript:

RSA	Repeated sprint ability
ATP-CP	Adenosine triphosphate-creatine phosphate system
PPO	Peak power output
MPO	Mean power output
BW	Body weight
PRISMA	Preferred Reporting Items for Systematic Evaluation and Meta-Analysis
PROSPERO	International Prospective Register of Systematic Reviews
SD	Standard deviation
WMD	Weighted mean difference
CI	Confidence interval
SE	Standard error
