# Supplementary material for: Effects of Acute Caffeine Ingestion on Repeated Sprint Ability: A Systematic Review and Meta-Analysis
Source: Nutrients. 2025 Nov 5;17(21):3475. doi: 10.3390/nu17213475 (PMC12608157; doi:10.3390/nu17213475)
Supplement: Supplementary file 1 [file nutrients-17-03475-s001.zip › Supplementary material.pdf]

Supplemental Material

Effects of acute caffeine ingestion on repeated sprint ability: a systematic review and meta-analysis

Figure S1. Results of Cochrane risk of bias tool...2

Figure S2. Funnel plot...3

Figure S3. Sensitivity analysis results...4

Table S1. Search strategies..5

Table S2. Characteristics of the studies included in this meta-analysis.........................8

Table S3. Methodological assessment of randomized controlled trials included in the systematic review using the PEDro scale...10

Table S4. Results of Egger's test..11

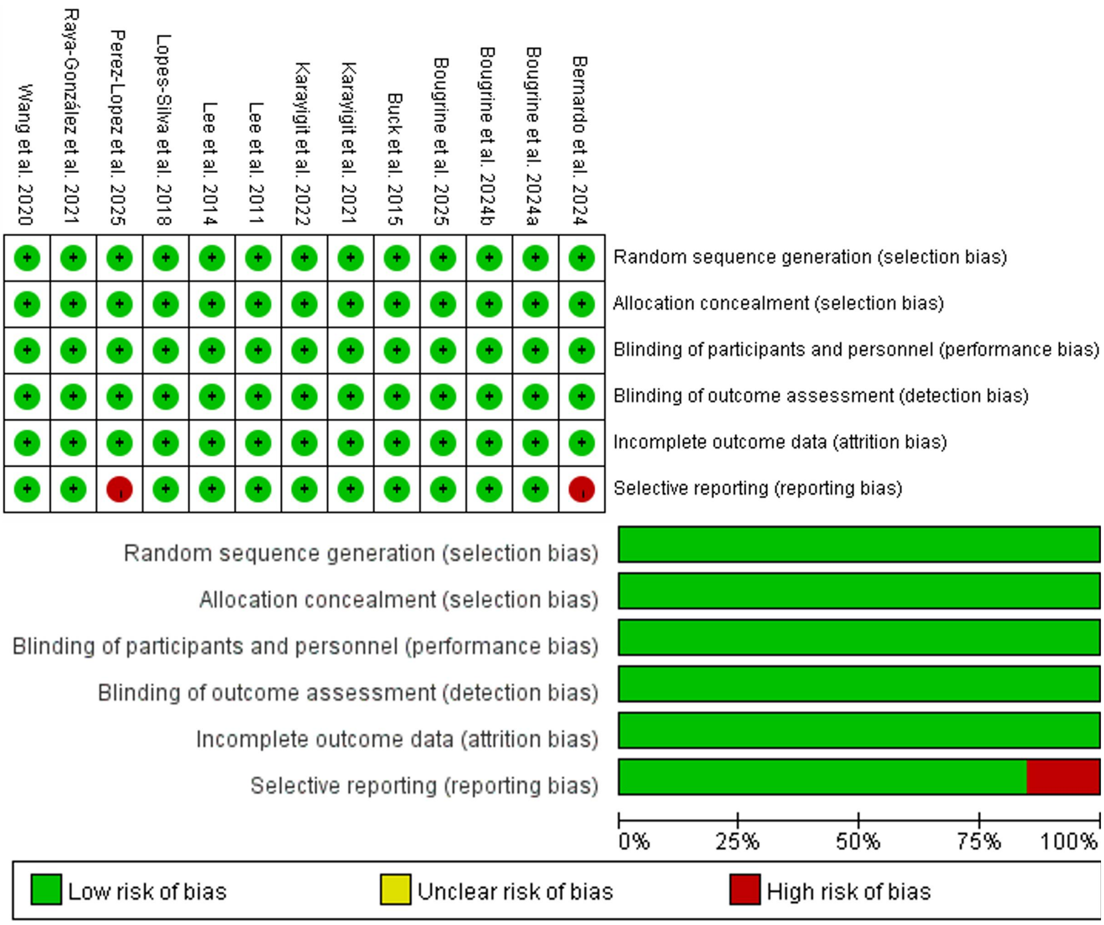

Figure S1. Results of Cochrane risk of bias tool.

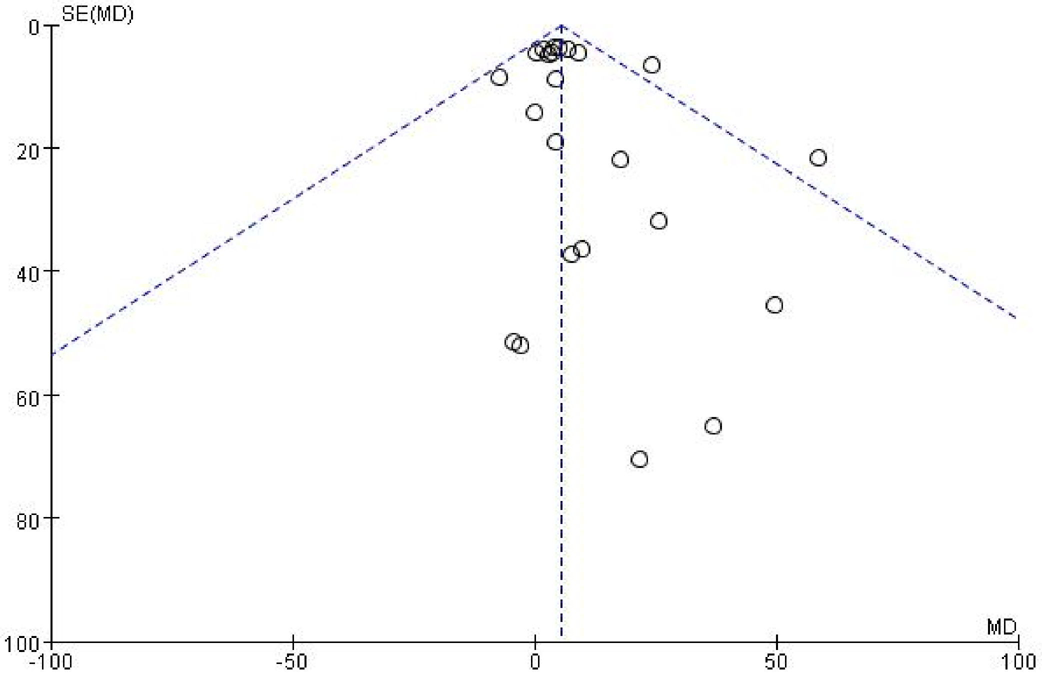

Figure S2. Funnel plot.

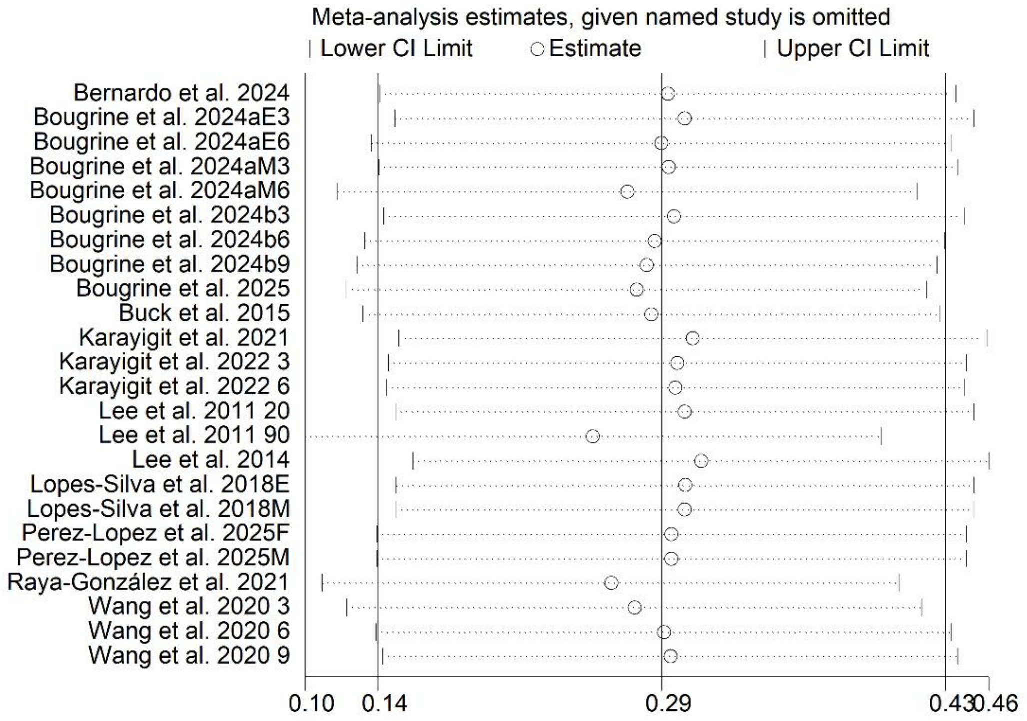

Figure S3. Sensitivity analysis results.

Table S1. Search strategies.

Web of Science

Term	Search	Query
Caffeine	#1	TS= ("Caffeine" OR "1,3,7-Trimethylxanthine" OR "No Doz" OR "Caffedrine" OR "Coffeinum N" OR "Coffeinum Purrum" OR "Dexitac" OR "Durvitan" OR "Percoffedrinol N" OR "Vivarin" OR "Percutaféine" OR "Quick-Pep" OR "QuickPep" OR "Quick Pep")
Repeated sprint	#2	TS= ("Repeated sprint" OR "Multiple sprint" OR "Intermittent sprint")
Combined	#3	#1 AND #2

PubMed

Term	Search	Query
Caffeine	#1	("Caffeine"[Mesh] OR " Caffeine"[tiab] OR " 1,3,7-Trimethylxanthine"[tiab] OR " No Doz"[tiab] OR "Caffedrine"[tiab] OR " Coffeinum N"[tiab] OR " Coffeinum Purrum"[tiab] OR "Dexitac"[tiab] OR "Percoffedrinol N"[tiab] OR "Vivarin"[tiab] OR "Percutaféine"[tiab] OR "Quick-Pep"[tiab] OR "QuickPep"[tiab] OR "Quick Pep"[tiab])
Repeated sprint	#2	("Repeated sprint"[Mesh] OR "Repeated sprint"[tiab] OR "Multiple sprint"[Mesh] "Multiple sprint"[tiab]OR "Intermittent sprint"[Mesh] OR "Intermittent sprint"[tiab])
Combined	#3	#1 AND #2

Cochrane Library

Term	Search	Query
Caffeine	#1	(MeSH descriptor: [Caffeine] explode all trees):ti,ab,kw
Repeated	#2	(MeSH descriptor: [Repeated sprint] explode all trees) OR

sprint		(repeated sprint OR "multiple sprint" OR "intermittent sprint"):ti,ab,kw
Combined	#3	#1 AND #2

Embase

Term	Search	Query
Caffeine	#1	'caffeine'/exp OR caffeine OR '1,3,7 trimethylxanthine'/exp OR '1,3,7 trimethylxanthine' OR 'no doz'/exp OR 'no doz' OR (('no'/exp OR no) AND doz) OR caffedrine OR 'coffeinum n' OR (coffeinum AND n) OR 'coffeinum purrum' OR (coffeinum AND purrum) OR dexitac OR durvitan OR 'percoffedrinol n' OR (('percoffedrinol'/exp OR percoffedrinol) AND n) OR 'vivarin'/exp OR vivarin OR percutaféine OR quickpep OR 'quick pep' OR (('quick'/exp OR quick) AND pep)
Repeated sprint	#2	'repeated sprint' OR (repeated AND ('sprint'/exp OR sprint)) OR 'multiple sprint' OR (multiple AND ('sprint'/exp OR sprint)) OR 'intermittent sprint' OR (intermittent AND ('sprint'/exp OR sprint))
Combined	#3	#1 AND #2

Scopus

Term	Search	Query
Caffeine	#1	(TITLE-ABS-KEY (QuickPep OR Quick Pep))OR (TITLE-ABS-KEY (Percutaféine OR Quick-Pep)) OR (TITLE-ABS-KEY (Percoffedrinol N OR Vivarin)) OR (TITLE-ABS-KEY (Durvitan)) OR (TITLE-ABS-KEY (Dexitac)) OR (TITLE-ABS-KEY (Coffeinum N)) OR

		(TITLE-ABS-KEY (caffedrine)) OR (TITLE-ABS-KEY (No Doz)) OR (TITLE-ABS-KEY (1 , 3 , 7-Trimethylxanthine)) OR (TITLE-ABS-KEY (caffeine))
Repeated sprint	#2	TITLE-ABS-KEY (Repeated sprint OR Multiple sprint OR Intermittent sprint)
Combined	#3	#1 AND #2

Table S2. Characteristics of the studies included in this meta-analysis.

Study	Sample size	Age (y)	Caffeine dose (mg/kg BW)	Timing of caffeine ingestion (min)	Exercise mode	Sprint protocol
Bernardo et al. 2024	12	26 ± 4	6	60	Cycling	12 × 6 s; 60 s rest
Bougrine et al. 2024a	15	18.3 ± 0.5	3, 6	60	Running	6 × (2 × 12.5 m) shuttle sprints; 20 s rest
Bougrine et al. 2024b	16	16.9 ± 0.6	3, 6, 9	60	Running	6 × (2 × 12.5 m) shuttle sprints; 20 s rest
Bougrine et al. 2025	17	16.7 ± 0.4	6	60	Running	6 × (2 × 12.5 m) shuttle sprints; 20 s rest
Buck et al. 2015	12	25.5 ± 1.9	6	60	Running	6 × 20 m; 25 s rest (only set 1)
Karayigit et al. 2021	24	22 ± 1.5	6	60	Cycling	12 × 4 s; 90 s rest
Karayigit et al. 2022	13	20 ± 1	3, 6	60	Cycling	12 × 4 s; 20 s rest
Lee et al. 2011	14	18.7 ± 0.8	6	60	Cycling	2 sets of 12 × 4 s; 20 s or 90 s rest between sprint, 4 min rest between sets
Lee et al. 2014	11	21.3 ± 1.2	6	60	Cycling	10 sets of 5 × 4 s; 20 s active

						recovery (60–70 rpm, 50 watts)
Lopes-Silva et al. 2018	13	26.4 ± 4	5	60	Cycling	10 × 6 s; 30 s rest
Perez-Lopez et al. 2025	52	24 ± 4.5	3	60	Cycling	4 × 30 s; 90 s rest
Raya-González et al. 2021	14	21 ± 2	6	60	Running	5 × 30 m; 30 s rest
Wang et al. 2020	10	20.88 ± 2.72	3, 6, 9	60	Cycling	4 × (15 × 5 s); 55 s rest

Note: y, year; BW, body weight; rmp, rotation per minute.

Table S3. Methodological assessment of randomized controlled trials included in the systematic review using the PEDro scale.

Study	A	B	C	D	E	F	G	H	I	J	K	Score
Bernardo et al. 2024	Y	1	1	1	1	1	1	1	1	1	1	10/10
Bougrine et al. 2024a	Y	1	1	1	1	1	1	0	1	1	1	9/10
Bougrine et al. 2024b	Y	1	1	1	1	1	1	0	1	1	1	9/10
Bougrine et al. 2025	Y	1	1	1	1	1	1	0	1	1	1	9/10
Buck et al. 2015	Y	1	1	1	1	1	1	1	1	1	1	10/10
Karayigit et al. 2021	Y	1	1	1	1	1	1	1	1	1	1	10/10
Karayigit et al. 2022	Y	1	1	1	1	1	1	1	1	1	1	10/10
Lee et al. 2011	Y	1	1	1	1	1	1	1	1	1	1	10/10
Lee et al. 2014	Y	1	1	1	1	1	1	1	1	1	1	10/10
Lopes-Silva et al. 2018	Y	1	1	1	1	1	1	1	1	1	1	10/10
Perez-Lopez et al. 2025	Y	1	1	1	1	1	1	1	1	1	1	10/10
Raya-González et al. 2021	Y	1	1	1	1	1	1	1	1	1	1	10/10
Wang et al. 2020	Y	1	1	1	1	1	1	1	1	1	1	10/10

Note: A, eligibility criteria; B, random allocation; C, concealed allocation; D, baseline comparability; E, blind subjects; F, blind therapists; G, blind assessors; H, adequate follow-up; I, intention-to-treat analysis; J, between-group comparisons; K, point estimates and variability. The total score represents the score of the PEDro scale. Item 1 was not scored. Y: yes.

Table S4. Results of Egger's test.

Std_Eff	Coef.	Std. Err.	t	p > |t|	95% CI
slope	-.02021941	0.493167	-0.41	0.686	-1.22496, 0.8205716
bias	1.333567	1.333231	1.00	0.328	-1.431385, 4.098519

Note: Coef, coefficient; Std. Err, standard error; t, t-test statistic; p, probability; CI, Confidence interval.
